# Dispersity within Brushes Plays a Major Role in Determining Their Interfacial Properties: The Case of Oligoxazoline-Based Graft Polymers

**DOI:** 10.1021/jacs.1c08383

**Published:** 2021-11-05

**Authors:** Matteo Romio, Benjamin Grob, Lucca Trachsel, Andrea Mattarei, Giulia Morgese, Shivaprakash N. Ramakrishna, Francesca Niccolai, Elisa Guazzelli, Cristina Paradisi, Elisa Martinelli, Nicholas D. Spencer, Edmondo M. Benetti

**Affiliations:** † Biointerfaces Lab, Swiss Federal Laboratories for Materials Science and Technology (Empa), Lerchenfeldstrasse 5, 9014 St. Gallen, Switzerland; ‡ Laboratory for Surface Science and Technology, Department of Materials, ETH Zürich, Vladimir-Prelog-Weg 5, 8093 Zürich, Switzerland; § George & Josephine Butler Polymer Research Laboratory, Department of Chemistry, University of Florida, P.O. Box 117200, Gainesville, Florida 32611-7200, United States; ∥ Department of Pharmaceutical and Pharmacological Sciences, University of Padova, Via Marzolo 5, 35131 Padova, Italy; ⊥ Institute of Materials and Process Engineering (IMPE), School of Engineering (SoE), Zürich University of Applied Sciences (ZHAW), Technikumstrasse 9, 8401 Winterthur, Switzerland; # Soft Materials and Interfaces, Department of Materials, ETH Zürich, Vladimir-Prelog-Weg 5, 8093 Zürich, Switzerland; ∇ Department of Chemistry and Industrial Chemistry, University of Pisa, Via Moruzzi 13, 56124 Pisa, Italy; ● Department of Chemical Sciences, University of Padova, Via Marzolo 1, 35122 Padova, Italy

## Abstract

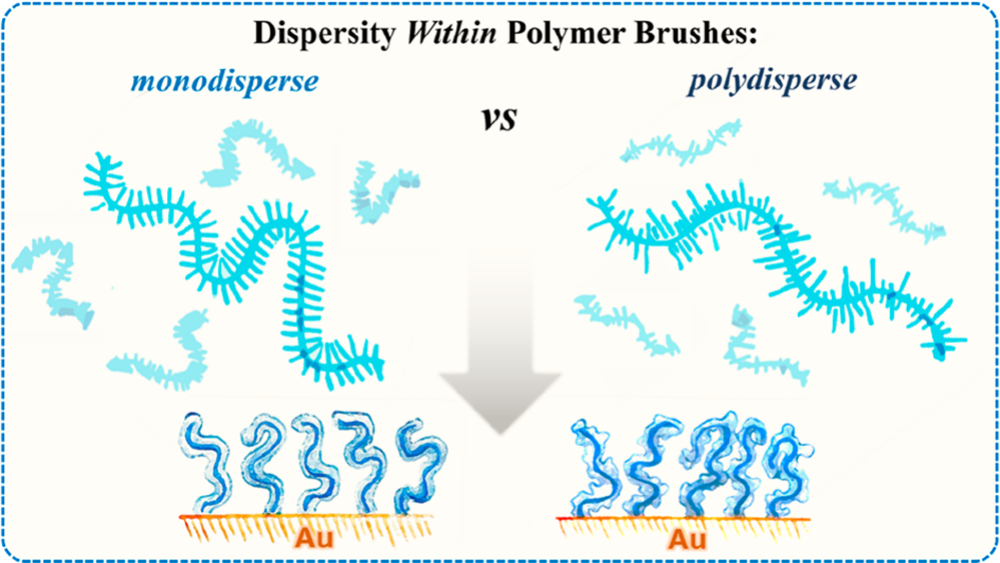

Many synthetic polymers used to form polymer-brush films feature a main backbone with functional, oligomeric side chains. While the structure of such graft polymers mimics biomacromolecules to an extent, it lacks the monodispersity and structural purity present in nature. Here we demonstrate that side-chain heterogeneity within graft polymers significantly influences hydration and the occurrence of hydrophobic interactions in the subsequently formed brushes and consequently impacts fundamental interfacial properties. This is demonstrated for the case of poly(methacrylate)s (PMAs) presenting oligomeric side chains of different length (*n*) and dispersity. A precise tuning of brush structure was achieved by first synthesizing oligo(2-ethyl-2-oxazoline) methacrylates (OEOXMAs) by cationic ring-opening polymerization (CROP), subsequently purifying them into discrete macromonomers with distinct values of *n* by column chromatography, and finally obtaining poly[oligo(2-ethyl-2-oxazoline) methacrylate]s (POEOXMAs) by reversible addition–fragmentation chain-transfer (RAFT) polymerization. Assembly of POEOXMA on Au surfaces yielded graft polymer brushes with different side-chain dispersities and lengths, whose properties were thoroughly investigated by a combination of variable angle spectroscopic ellipsometry (VASE), quartz crystal microbalance with dissipation (QCMD), and atomic force microscopy (AFM) methods. Side-chain dispersity, or dispersity *within* brushes, leads to assemblies that are more hydrated, less adhesive, and more lubricious and biopassive compared to analogous films obtained from graft polymers characterized by a homogeneous structure.

## Introduction

Controlled polymerization techniques, and especially reversible-deactivation radical polymerizations (RDRPs), have progressively enabled the synthesis of narrowly dispersed macromolecules under extremely accessible conditions.^1^ Very recently, the fine-tuning of reaction conditions during RDRP has additionally permitted the precise modulation of molecular weight dispersity (*Đ*) over a relatively broad range of values.^2,3^

Besides this representing a fundamental advance in the synthesis of polymers, recent developments in the control over *Đ* are now revitalizing the interest of polymer and materials scientists, who are trying to determine the impact that any heterogeneity in size and/or molecular architecture of polymer components might have on the physicochemical properties of derived materials.^4^

Extensive work has been conducted in elucidating the role that dispersity plays in determining the morphology of nanostructured materials obtained from self-assembly of block polymers, both in solution^5−7^ and in bulk.^8−10^ More recently, increased interest in the effects that a variation of *Đ* could have on the properties of oligomeric species has been developing because of the fundamental studies by Hawker et al.^11−14^ and Meijer et al.,^15−19^ who have explored the morphological and structural characteristics of a variety of self-assembled nanostructures derived from block co-oligomers.

Despite the evident blooming of research in this particular field, very little has been reported about the relationship between dispersity and the properties of polymer interfaces, or polymer nanoassemblies at solid surfaces.

Focusing on polymer brushes, Matyjaszewski and Bockstaller have highlighted that when polymer brushes are grafted from silica nanoparticles (NPs), their grafting density (σ) and *Đ* determine the morphology of the subsequently obtained hybrids. Narrowly dispersed brushes enabled the formation of uniform films of NPs, while broadly dispersed grafts generated anisotropic structures presenting string-like NP aggregates.^20^ These structural differences were later demonstrated to affect the mechanical properties of brush-particle-composite films.^21^

Alternatively, while applying polymer brushes as biointerfaces, Yadav et al. demonstrated that by increasing the molar mass and *Đ* of polyacid films on macroscopic surfaces, a fully reversible attachment of bacteria could be achieved by switching pH of the medium.^22^

These initial studies seem to suggest that in addition to polymer composition, molar mass, and surface coverage, *Đ* could also represent an additional tuning parameter for modulating the interfacial characteristics of polymer brushes.

However, these reports uniquely investigated a modulation in *main-chain dispersity* and its effects on materials properties. In contrast, a large variety of synthetic polymer brushes includes a polymer backbone and functional, oligomeric (or polymeric) side chains, with a design that is reminiscent of the structure of biopolymers, such as proteoglycans,^23^ which, it should be noted, are intrinsically characterized by discrete, *monodisperse* structures^24^ and provide distinctive properties and functions to different tissues within our body.^25^

**Scheme 1:**
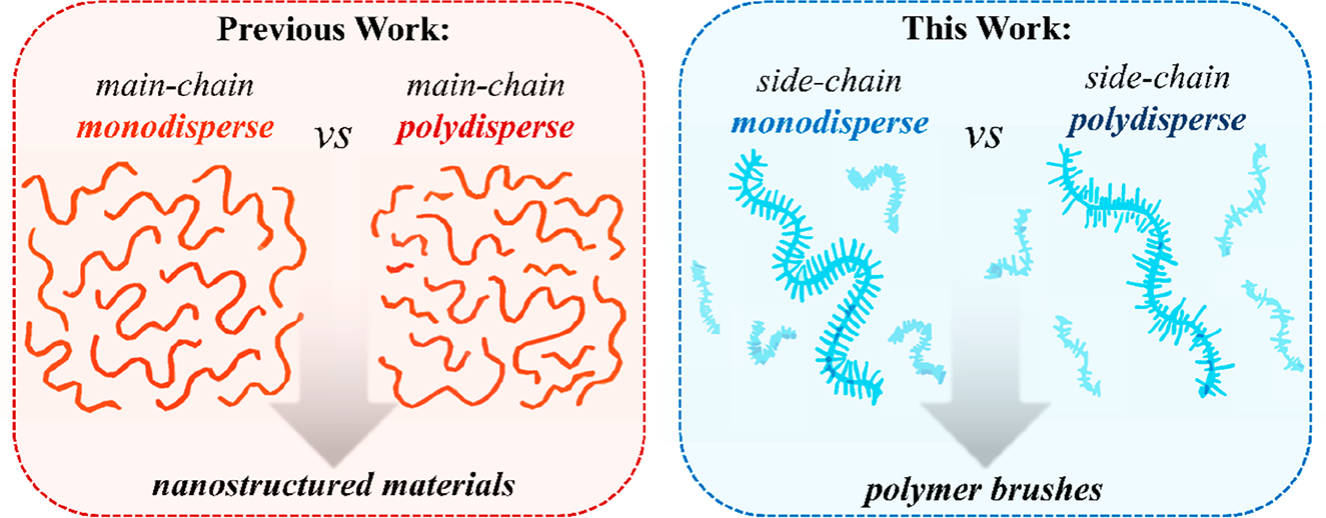
While in previous work the effect of main-chain dispersity of (co)polymers on the properties of subsequently derived materials was investigated, side-chain dispersity lies in the focus of the present study.

Hence, dispersity of side chains (or *side-chain heterogeneity*) within polymer brushes emerges as a possible tuning parameter for an array of technologically relevant, interfacial physicochemical properties and lies at the focus of the present study (Scheme 1).

More generally, the impact of variation in the degree of heterogeneity of grafted polymer architectures on essential properties such as steric stabilization of the substrate and amount of coupled solvent has yet to be demonstrated. These parameters directly determine fundamental characteristics of brush coatings, such as their resistance toward nonspecific biological contamination,^26^ their lubricious properties,^27,28^ or colloidal stabilization,^29,30^ when they are grafted from NPs.

In this study, the effect of *side-chain dispersity* of polymer brushes assembled on flat substrates is specifically investigated for the case of graft polymers constituted by poly(methacrylate)s (PMAs) presenting oligomeric side chains. Here, these are based on poly[oligo(2-ethyl-2-oxazoline) methacrylate]s (POEOXMAs),^31−36^ which were synthesized by reversible addition–fragmentation chain transfer (RAFT) polymerization of oligo(2-ethyl-2-oxazoline) methacrylates (OEOXMAs), previously obtained by cationic ring-opening polymerization (CROP) of 2-ethyl-2-oxazoline (EOX).

Graft polymers with similar structures, such as poly[oligo(ethylene glycol) methacrylate] (POEGMA), are widely applied in materials science for the functionalization of biomaterials,^37,38^ the fabrication of biosensors^39^ and thermoresponsive coatings,^40,41^ and in a broad range of other applications.^42−45^ In addition, they are considered to be “standards” for performing RDRPs in aqueous media.^46−50^ However, despite their widespread use, POEGMAs usually feature a distribution of OEG_*n*_ side-chain lengths (*n*), which is typically centered at *n* ≈ 10,^42^ although this value might vary across different monomer batches and sources. In other words, POEGMA brushes applied in some of the most common materials formulations and coatings are characterized by an intrinsic dispersity in their side-chain length, or a certain degree of heterogeneity in their structure, with side chains that do not feature a well-defined *n*.

In order to investigate how the side-chain length and dispersity in the brush structure influence their interfacial properties, we concentrated on POEOXMA, as 2-oxazoline-based polymers represent some of the most promising replacements for PEG and its derivatives in biomaterials and biotechnology.^51−54^ Precise tuning of graft polymer brush structure was achieved by first synthesizing OEOXMAs, which are intrinsically polydisperse, and subsequently purifying them into discrete macromonomers with distinct *n* by column chromatography.^11−13^

Polydisperse OEOXMA and monodisperse macromonomers with different *n* values were subsequently polymerized by RAFT polymerization, and the obtained POEOXMAs were finally derivatized with a disulfide-based anchor, enabling their assembly on Au surfaces to yield brush nanofilms.

The properties of chemically similar but structurally different POEOXMA brushes were characterized by a variety of surface-analytical methods. Assembly of brushes, their hydration, steric stabilization, nanotribological and antifouling properties were investigated by a combination of quartz crystal microbalance with dissipation (QCMD), variable-angle spectroscopic ellipsometry (VASE), and atomic force microscopy (AFM)-based methods.

Results from these approaches demonstrate how the presence of polydisperse side chains leads to an increase in hydration, in comparison to graft polymer brushes incorporating monodisperse oligomers within each monomer unit. Enhanced hydration simultaneously leads to an improvement in several relevant properties, including lubrication and biopassivity. In contrast, the presence of side chains with a well-defined length within brushes with a homogeneous structure favors the occurrence of hydrophobic interactions, markedly degrading both lubricious properties and resistance toward nonspecific protein contamination.

Overall, *side-chain dispersity* within nanofilms of graft polymer brushes emerges as a major factor in their propensity to hydrate, in addition to well-known parameters, such as composition and molar mass. Especially in the case of brushes presenting oligomeric side chains, or graft polymer brushes, which are commonly applied in myriad formulations, this parameter is revealed to have significant impact on interfacial physicochemical properties.

## Results and Discussion

**Figure 1:**
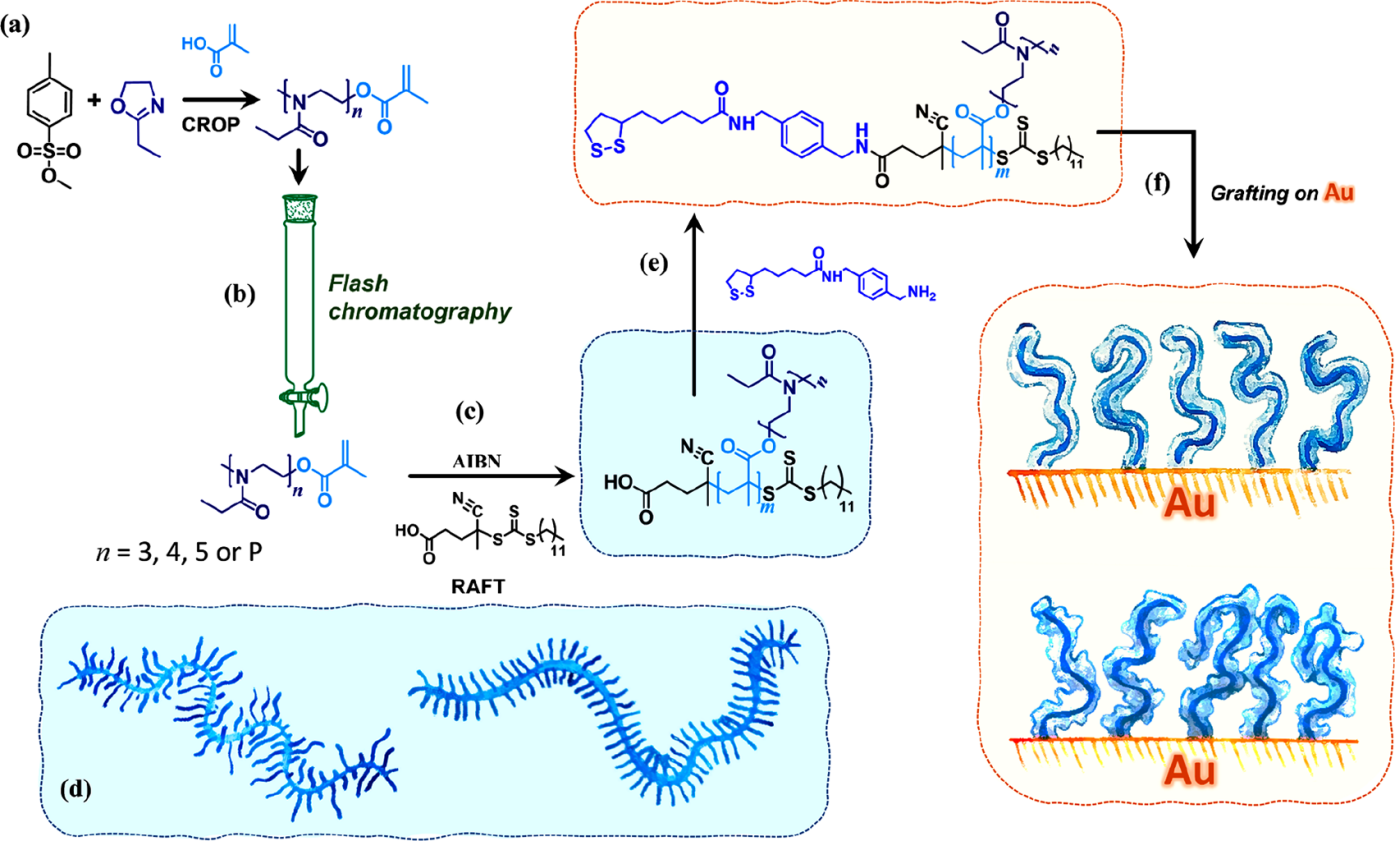
CROP using MeOTs as initiator and MA as terminator agent provided unfractionated OEOX_P_MA (a), which could be subsequently purified by flash column chromatography yielding monodisperse OEOX_*n*_MA (b). OEOX_*n*_MA, with *n* = 3, 4, and 5, and unfractionated OEOX_P_MA were subsequently polymerized by RAFT (c), using AIBN as radical initiator and CDPA as chain-transfer agent, finally obtaining POEOXMAs-COOH with different dispersity and length *n* of side chains (d). Coupling of ANPIS to POEOXMAs-COOH provided graft polymer adsorbates that could be assembled (e) from ethanolic solution onto Au substrates forming brushes (f).

**Figure 2:**
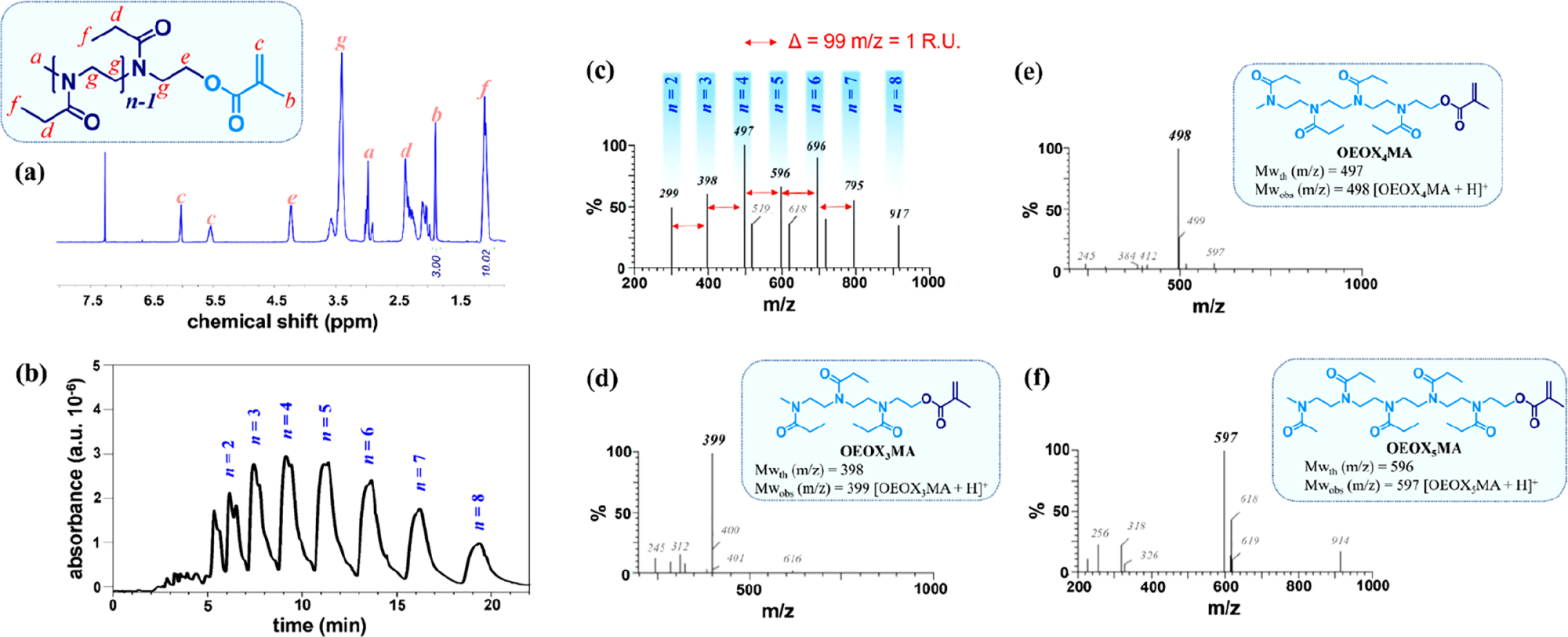
(a) ^1^H NMR spectrum of polydisperse OEOXMA obtained by CROP. (b) HPLC elugram of polydisperse OEOXMA highlighting the different OEOX_*n*_MA fractions. Liquid chromatography–mass spectrometry (LC–MS) spectra of (c) polydisperse OEOXMA, (d) OEOX_3_MA, (e) OEOX_4_MA, and (f) OEOX_5_MA.

Structurally different POEOXMAs were molecularly designed starting from their monomeric constituents. OEOXMAs were initially synthesized by CROP using methyl *p*-toluenesulfonate (MeOTs) as initiator and methacrylic acid (MA) as terminator agents (Figure 1a). The resulting OEOX_*n*_MA featured an average *n* of 3.3, as measured by ^1^H nuclear magnetic resonance (^1^H NMR) spectroscopy (Figure 2a). However, *n* was intrinsically characterized by a rather broad distribution of values, as evidenced by high-performance liquid chromatography (HPLC) (Figure 2b). Polydisperse OEOX_*n*_MA (which is identified as OEOX_P_MA) could be purified into monodisperse fractions presenting a well-defined *n* by flash column chromatography (Figure 1b and Methods). This process gave access to OEOX_*n*_MA presenting *n* = 3, 4, and 5 (OEOX_3_MA, OEOX_4_MA and OEOX_5_MA, respectively), which are the most abundant fractions present within OEOX_P_MA (Figure 2b–f).

Subsequent RAFT polymerization of OEOXMAs provided POEOXMA species with different degrees of heterogeneity and lengths of oligomeric side chains (Figure 1c,d).

As reported in Table 1, using 4-cyano-4-[(dodecylsulfanylthiocarbonyl)sulfanyl]pentanoic acid (CDPA) as a RAFT agent and azobisisobutyronitrile (AIBN) as a radical initiator, all the different OEOXMAs could be polymerized, reaching conversions higher than 90% in 24 h and yielding carboxylic acid-terminated POEOXMAs (POEOXMAs-COOH, Figure 1c) with *Đ* lying between 1.15 and 1.35 (Table 1).

**Table 1:** Characterization of POEOXMA Adsorbates and The Corresponding Brushes

polymer	*M*_n_ (SEC) [kDa]	Đ	*M*_n_ (NMR) [kDa]	*T*_dry_ (VASE) [nm]	*T*_wet_ (QCM) [nm]	*W* [%]	σ^a^ [nm^-2^]	θ_S_ [deg]	θ_A_ [deg]	θ_R_ [deg]
POEOX_P_MA	10.8	1.21	22.8	3.0 ± 0.1	7.1 ± 0.1	58 ± 3	0.10 ± 0.01	39.6 ± 0.6	41.3 ± 0.5	23.3 ± 0.7
POEOX_3_MA	11.5	1.35	25.1	3.1 ± 0.1	6.2 ± 0.3	49 ± 8	0.09 ± 0.01	42.9 ± 2.1	44.0 ± 0.8	28.5 ± 1.7
POEOX_4_MA	15.6	1.32	24.9	3.2 ± 0.2	6.4 ± 0.1	51 ± 5	0.09 ± 0.01	44.8 ± 1.0	46.4 ± 1.1	25.5 ± 1.6
POEOX_5_MA	20.9	1.15	16.1	2.7 ± 0.1	5.7 ± 0.1	52 ± 4	0.11 ± 0.01	41.9 ± 2.2	44.3 ± 1.8	25.6 ± 1.7

a Grafting density (σ), expressed as [chains nm^–2^], was calculated using the equation σ = ρ*T*_dry_*N*_A_*M*_n_^–1^ where ρ is the density of the dry polymer layer (1.14 g cm^–3^), *T*_dry_ is the dry thickness measured by VASE, *N*_A_ is the Avogadro number, and *M*_n_ is the average molar mass of the adsorbate measured by ^1^H NMR.

Subsequent conjugation of POEOXMAs-COOH with *N*-(4-(aminomethyl)benzyl)-5-(1,2-dithiolan-3-yl)pentanamide (ANPIS) provided graft polymer adsorbates with a disulfide end group (named as POEOX_P_MA-, POEOX_3_MA-, POEOX_4_MA-, and POEOX_5_MA-ANPIS) that could function as an anchor for their subsequent assembly on Au surfaces (Figure 1e,f).

Assembly of POEOXMAs-ANPIS on Au and the formation of the corresponding brushes were performed by incubation of freshly cleaned Au-coated silicon substrates in 1 mg mL^–1^ ethanolic solutions of the different polymer adsorbates, followed by extensive rinsing with ethanol and ultrapure water.

The properties of POEOXMA brushes in the dry state were monitored by ex situ VASE and in the swollen state by in situ QCMD.

As shown in Table 1, following 1 h of adsorption, the different POEOXMA-based adsorbates formed brush layers with dry thicknesses (*T*_dry_) ranging from 2.7 to 3.2 nm, as measured by VASE, corresponding to grafting densities (σ) that are similar for the different assemblies, lying in the range of 0.09–0.11 chains nm^–2^.

Despite their very similar values of *T*_dry_ and σ, interestingly, different POEOXMAs-ANPIS generated brushes with diverse swelling properties, which could be evaluated by QCMD.

It is particularly instructive to compare assembly and hydration of brushes obtained via chemisorption of POEOX_3_MA-ANPIS and POEOX_P_MA-ANPIS onto Au surfaces. These two graft polymer adsorbates present very similar values of *M*_n_ and comparable, as obtained by size-exclusion chromatography (SEC) and confirmed by ^1^H NMR (Table 1), and thus they feature similar values of both number average degree of polymerization and main-chain dispersity. However, POEOX_3_MA-ANPIS and POEOX_P_MA-ANPIS are characterized by side OEOX chains with markedly different length distributions. POEOX_3_MA presents monodisperse, oligomeric side chains with *n* = 3, whereas POEOX_P_MA is characterized by broadly dispersed side chains with a maximum in the distribution of *n* lying between 3 and 4, as inferred by HPLC and ^1^H NMR (Figure 2a,b). Hence POEOX_3_MA and POEOX_P_MA are illustrative of two polymers presenting similar main-chain characteristics but a dissimilar degree of structural heterogeneity because of a markedly different dispersity of their side chains.

**Figure 3:**
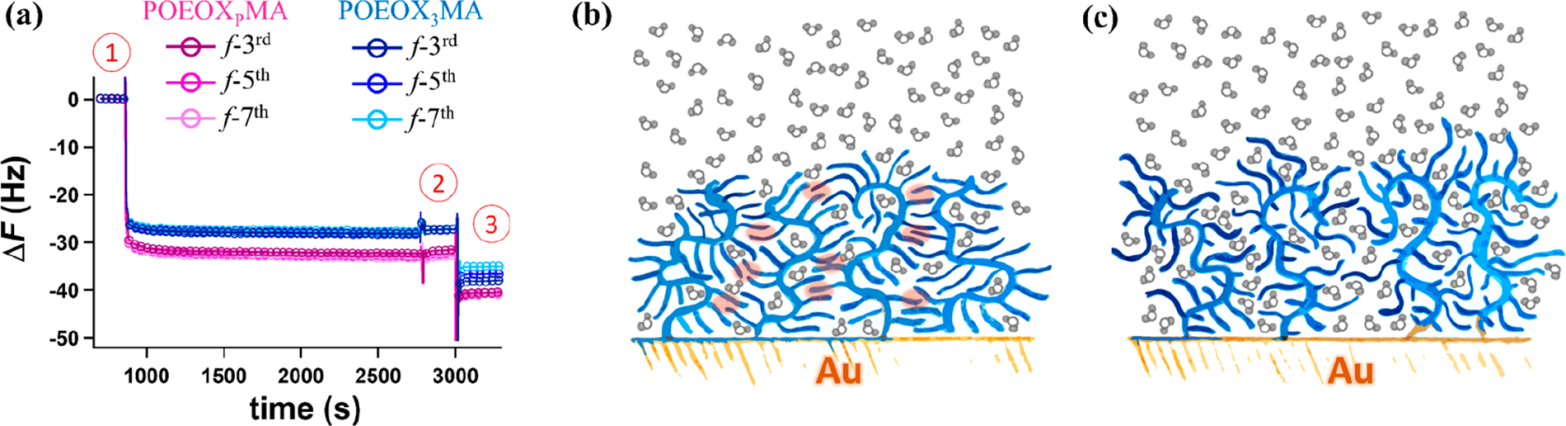
(a) QCMD sensograms reporting the variation of Δ*F* during the assembly of POEOX_3_MA and POEOX_P_MA brushes on Au-coated sensors. Three different overtones (*f*-3^rd^, *f*-5^th^, and *f*-7^th^) were reported for each sample. Point 1 corresponds to injection of polymer solutions. At point 2 the formed brushes were rinsed by injecting pure ethanol, and at point 3 flushing with ultrapure water was carried out. Fitting of the *f*-3^rd^, *f*-5^th^, and *f*-7^th^ overtones by an extended viscoelastic model provided the values of *T*_wet_ for POEOX_3_MA and POEOX_P_MA brushes. (b, c) Illustrations highlighting the less hydrated assembly of POEOX_3_MA grafts (b), in which hydrophobic interactions between chains are taking place, and the enhanced hydration of POEOX_P_MA brushes (c).

Typical QCMD sensograms reporting the variation in frequency (Δ*F*) for three different overtones (*f*-3^rd^, *f*-5^th^, and *f*-7^th^) while assembling POEOX_3_MA-ANPIS and POEOX_P_MA-ANPIS on Au are reported in Figure 3a.

In both cases, the polymers readily assembled on Au, showing a rapid chemisorption until a plateau was reached, a few minutes after the injection of the polymer solutions.

Although the two different brushes presented similar values of *T*_dry_, the assembly of POEOX_P_MA-ANPIS on Au was mirrored by a larger Δ*F*, compared to that recorded during the assembly of POEOX_3_MA-ANPIS. A larger value of Δ*F* at similar *T*_dry_ necessarily implies a higher amount of coupled water.^55,56^

The values of swollen thickness (*T*_wet_) could be obtained by fitting the Δ*F* and dissipation data (Δ*D*) with an extended viscoelastic model,^56,57^ yielding *T*_wet_ = 6.2 ± 0.5 and 7.1 ± 0.1 nm for POEOX_3_MA and POEOX_P_MA brushes, respectively. These values were used to estimate the water content (calculated as *W* % = [(*T*_wet_ – *T*_dry_)/*T*_wet_] ×100 for each brush type, which yielded ∼49% for POEOX_3_MA and ∼58% for POEOX_P_MA brushes. Hence, polymer brushes presenting polydisperse side chains showed higher hydration when compared to chemically identical grafts of similar grafting densities, featuring dicrete oligomers in their monomer units (Figure 3b,c).

When comparing POEOXMA brushes with *monodisperse* OEOX side chains of different lengths, an increment in *n* from 3 to 5 was mirrored by a slight but progressive increase in *W* % (Table 1). However, even POEOX_5_MA brushes (featuring the longest OEOX segments among the studied assemblies) showed a lower swelling with respect to brushes presenting a high degree of heterogeneity in their side-chain length, which averaged only 3.3.

These results suggested that the surface concentration of hydrophilic units^58^ is not the only parameter controlling hydration of brushes and that side-chain dispersity appears to be a major factor.

The higher hydration tendency of POEOX_P_MA brushes was further confirmed by static and dynamic contact angle (CA) measurements.

As reported in Table 1, POEOX_P_MA brushes were characterized by the lowest value of static contact angle (θ_S_), which was 39 ± 0.6°. For monodisperse side chains, higher contact angles were observed, with θ_S_ = 43 ± 2°, 45 ± 1°, and 42 ± 2° for POEOX_3_MA, POEOX_4_MA, and POEOX_5_MA brushes, respectively. Dynamic wettability analysis confirmed the higher affinity of POEOX_P_MA brushes toward water. Advancing and receding contact angle (θ_A_ and θ_R_, respectively) values recorded on POEOX_P_MA brushes were 41 ± 1° and 23 ± 1°, respectively. In contrast, θ_A_ values were included between 44° and 46° for POEOXMA brushes with monodisperse OEOX, whereas the corresponding θ_R_ were in all cases ≥26° (Table 1).

**Figure 4:**
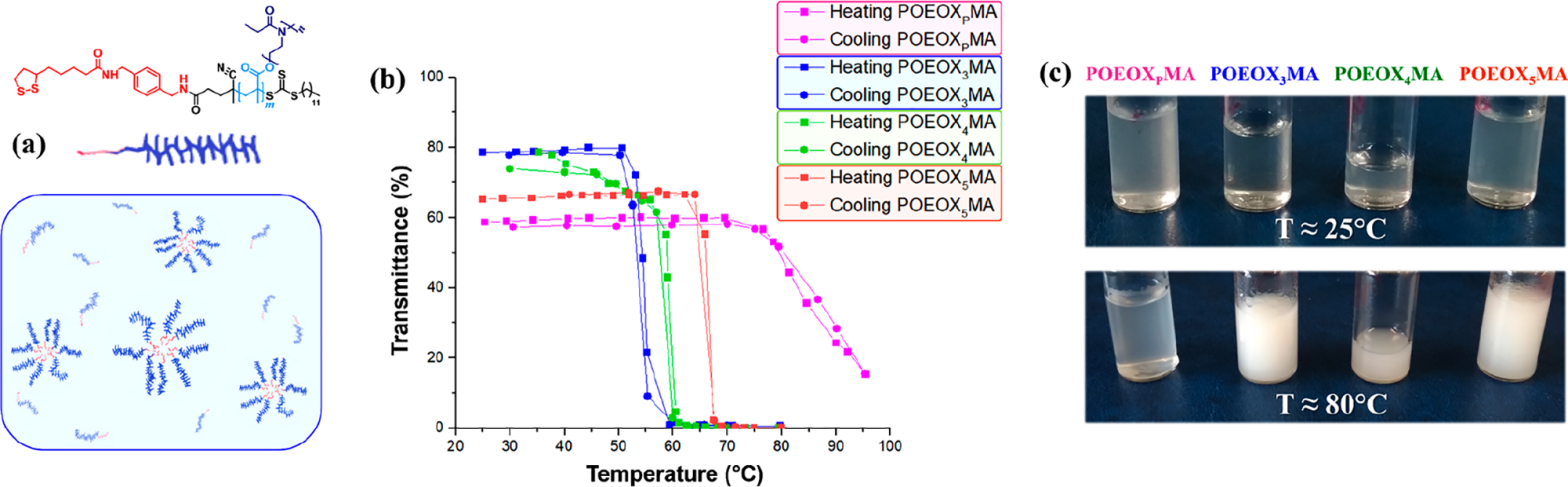
(a) POEOXMA adsorbates form micellar dispersions in aqueous media due to the presence of hydrophobic ANPIS anchors. (b) Turbidity curves recorded by UV/vis on 5 mg mL^–1^ dispersions of POEOXMA in ultrapure water. (c) Dispersions of the different POEOXMA adsorbates at ∼25 °C and after heating at ∼80 °C.

The different tendency to hydrate by POEOXMA adsorbates featuring diverse side chain-dispersity was further confirmed by analyzing their thermoresponsive properties in water (Figure 4).

Due to the presence of hydrophobic ANPIS anchor groups, all POEOXMA adsorbates formed stable micellar dispersions in solution (Figure 4a). However, the obtained dispersions were all characterized by a lower critical solution temperature (LCST), in accordance to previous studies focusing on compositionally similar OEOX-based graft polymers.^31^ Relevantly, turbidity measurements performed by UV/vis spectroscopy highlighted a significantly higher value of cloud point temperature (CP) for POEOX_P_MA dispersions, which was centered at ∼85 °C (Figure 4b,c). In contrast, analogous dispersions of POEOXMA adsorbates with side chains of discrete length showed significantly lower values of CP, which were 67°, 59°, and 55 °C, for POEOX_5_MA, POEOX_4_MA, and POEOX_3_MA, respectively.

Hence, enhanced hydration by POEOX_P_MA adsorbates, which was clearly significant in the case of micellar dispersions, translated into an increment in water uptake by analogous surface-grafted assemblies that was less remarkable. However, the differences in hydration that could be visualized by QCMD/VASE, and the intrinsically different structure of POEOX_P_MA brushes with respect to POEOXMA brush analogues presenting monodisperse side segments translated into significant variations in an array of technologically relevant interfacial properties (*vide infra*).

The increased hydration capability by POEOXMA adsorbates with polydisperse side chains could be generally explained by considering the effect of intramolecular and intermolecular side-chain interactions (hydrophobic/van der Waals) when amphiphilic grafts are confined to flat, macroscopic surfaces within a densely grafted brush assembly (Figure 3b,c) (or when these form the shell of a micellar structure, as displayed in Figure 4).

An increase in hydrophobic effects within amphiphilic brushes as a function of surface coverage was previously described by Schwartz *et al.* in the case of linear PEG grafts.^59^ Generally, an increase in σ leads to two distinct effects on the interfacial properties of PEG brushes. On the one hand, an increment in surface density leads to an increase in steric stabilization of the surface through osmotic and entropic effects.^26,60^ On the other hand, an increase in grafted-chain crowding favors the occurrence of hydrophobic interactions between nonionic, amphiphilic segments.^59,61^

POEOXMA brushes present amphiphilic side chains at each monomer unit and hydrophobic backbones based on PMA chains. Hence, hydrophobic effects involving OEOX segments and PMA backbones are likely to arise both intramolecularly, within the same graft, and intermolecularly, *i.e.*, between neighboring POEOXMA chains. The extent of such hydrophobic effects and how they interfere with the association of water molecules within POEOXMA brushes must be correlated to the length and dispersity of their side OEOX chains, as these two parameters are expected to determine interactions within the polymer structure (between neighboring side segments or involving side segments and PMA backbone) and between neighboring surface-grafted chains.

Besides wettability, the occurrence of hydrophobic interactions influences several interfacial properties of polymer brushes, including adhesion, lubrication, and resistance toward nonspecific contamination by serum proteins.^52,62^

In order to elucidate the relationship among interfacial properties, hydrophobic effects, and side-chain dispersity, we analyzed the different POEOXMA brushes by colloidal probe microscopy (CPM) and lateral force microscopy (LFM). During CPM and LFM, 20 μm diameter Au-coated silica particles, which were previously functionalized with the same POEOXMA brushes that were assembled on Au substrates, were used as probes.

**Figure 5:**
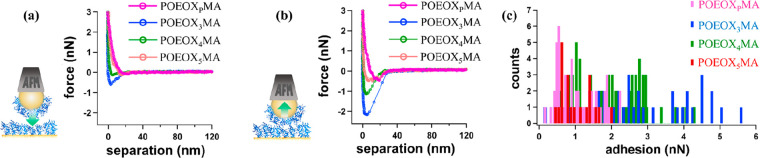
(a) Representative approach curves from FS profiles recorded by CPM on POEOXMA brushes and (b) the corresponding retract FS curves. As depicted in the insets, in each experiment an AFM colloidal probe bearing a POEOXMA brush identical to that deposited on the Au surface was employed. (c) Measured pull-off force distributions recorded by compressing the different POEOXMA brushes with identical brush countersurfaces.

For all POEOXMA brushes, force-vs-separation (FS) profiles were characterized by approach curves showing “snapping in” at separations of ≤20 nm, due to net attractive forces between opposing brush surfaces (Figure 5a). Simultaneously, retract curves were characterized by adhesive interactions, which were generally due to van der Waals forces taking place between POEOXMA brush surfaces compressed against each other (Figure 5b).^52^ However, adhesion was relatively low in the cases of POEOX_P_MA and POEOX_5_MA brushes, whereas significantly higher values were recorded for POEOX_4_MA and especially POEOX_3_MA (Figure 5c).

Generally, adhesion between opposing POEOXMA brush films was determined by hydrophobic interactions and hydration. As previously mentioned, hydrophobic interactions could occur both intramolecularly, between neighboring OEOX side chains as well as involving PMA backbones, and intermolecularly between side segments and main chains by polymers that are grafted close to each other on the Au surface.

In the case of POEOX_5_MA brushes, the presence of longer, OEOX side chains favored hydration and reduced the occurrence of hydrophobic interactions between PMA backbones. In contrast, within POEOX_P_MA brushes side-chain dispersity appeared to be an additional, major factor in determining adhesion, as the presence of polydisperse side chains determined an increment in hydration and simultaneously hindered hydrophobic interactions within brushes. As a result of the combination of both these effects, POEOX_P_MA brushes showed the lowest values of pull-off force among the studied samples.

**Figure 6:**
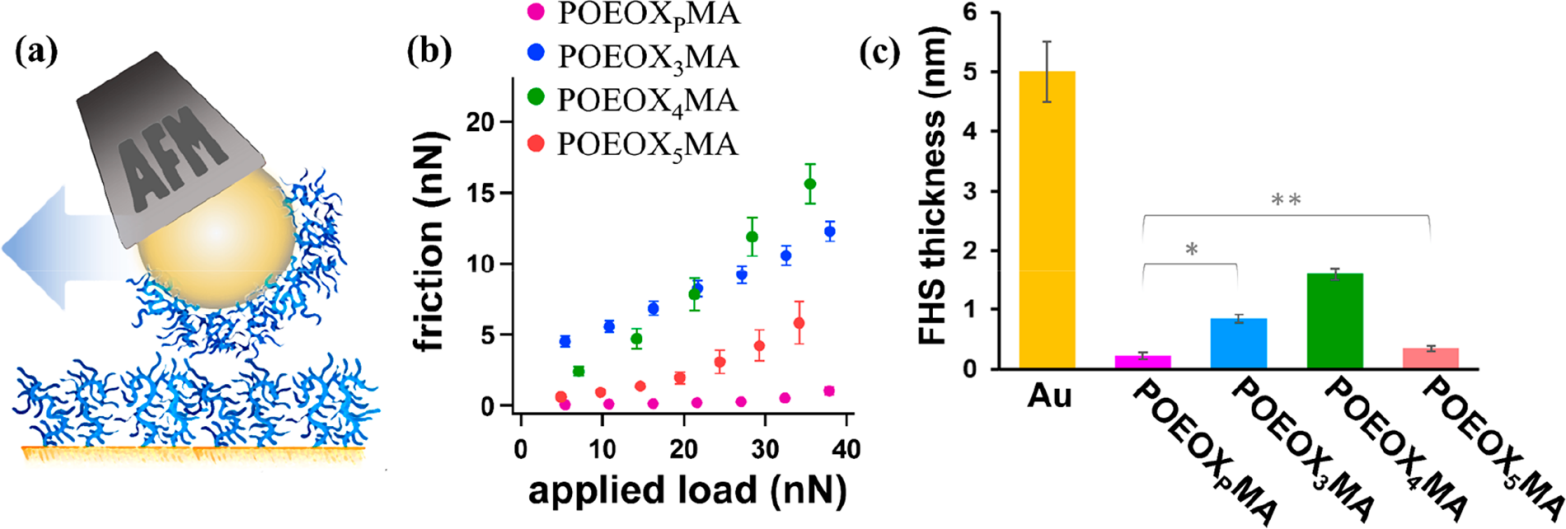
(a) LFM was performed by shearing a POEOXMA brush-functionalized colloidal AFM probe against an identical brush countersurface. (b) F_f_L profiles recorded for the different POEOXMA brush tribopairs. (c) Thickness of FHS recorded by VASE after 1 h of incubation of POEOXMA brushes, where the bare Au surface was used as control. * and ** indicate statistically significant differences between the set of data (*p < 0.01; **p < 0.05).

A similar trend was recorded while comparing the nanotribological properties of POEOXMA brushes. These were analyzed by LFM, shearing an Au-coated colloidal AFM probe functionalized with POEOXMA brushes over an Au substrate presenting identical grafts (Figure 6a). As highlighted in the friction force-vs-applied load profiles (F_f_L) reported in Figure 6b, POEOX_P_MA brushes demonstrated the most lubricious assemblies among the studied brushes, with a coefficient of friction (μ) approximating 0.02. In contrast, POEOX_3_MA and POEOX_4_MA grafts were characterized by significantly higher friction, with an order of magnitude higher values of μ, which were 0.25 and 0.35, respectively, while just a slight increase in lubrication was attained for POEOX_5_MA brushes, which showed μ = 0.17.

Hence, CPM and LFM analyses showed that POEOX_P_MA brushes, incorporating heterogeneous side chains, are more hydrated, less adhesive, and significantly more lubricious with respect to analogous graft polymer brushes comprising monodisperse OEOX segments. An increase in side-chain length, as in the case of POEOX_5_MA brushes, also led to a suppression of van der Waals interactions and a decrease in friction. Nevertheless, side-chain dispersity appeared as a crucial parameter in determining nanomechanical and nanotribological properties of POEOXMA brushes.

Enhanced hydration by graft polymer brushes featuring polydisperse side chains also influenced their resistance toward nonspecific protein contamination. In particular, the different POEOXMA brushes were incubated for 1 h in full human serum (FHS), and the amount of physisorbed proteins was subsequently evaluated by VASE (Figure 6c).

When comparing brushes incorporating monodisperse OEOX side segments, the lowest amount of adsorbed proteins was recorded on POEOX_5_MA brushes, which present the longest side OEOX chains. This result agreed well with the typical biopassive behavior of PAOXA and PEG brush surfaces, according to which a significant increase in surface concentration of ethylene glycol (EG) or alkyloxazoline (AOX) units was mirrored by a concomitant, relevant decrease in the mass of adsorbed serum proteins.^58,63^

The unexpected and somewhat counterintuitive properties of POEOX_4_MA brushes are worth pointing out, since this polymer showed the highest thickness of physisorbed proteins and the highest values of μ, compared to both POEOX_3_MA and POEOX_5_MA analogues. This results from the combination of two effects, side-chain/main-chain and side-chain/side-chain hydrophobic interactions, only one of which is dominant for POEOX_3_MA and POEOX_5_MA, respectively. However, in the case of the intermediate-length POEOX_4_MA both effects are apparently in operation.

In good agreement with the results from the adhesion and friction tests, the best protein repellency was recorded in the case of POEOX_P_MA brushes, on which a protein layer with a thickness of just ∼0.2 nm was formed, nearly 4-fold thinner than that recorded on POEOX_3_MA and 8-fold thinner than that formed on POEOX_4_MA.

Hence, the capability to associate water molecules and thus to provide an energetic barrier against nonspecific interactions with proteins depends not only on the surface density of biopassive units within polymer brushes but also on their side-chain dispersity. This parameter strongly influences the occurrence of hydrophobic interactions between surface-grafted chains, especially in the case of bioinert but amphiphilic brushes. In other words, side-chain dispersity of brushes seems to be acting in a similar way to surface dilution^59^ or brush mixing,^61^ which were both previously demonstrated to enhance water uptake.

## Conclusions

In this study, the impact of *side-chain dispersity* within graft polymer brushes on a set of interfacial properties was investigated through a variety of surface analytics.

Generally, the presence of polydisperse OEOX side chains within POEOXMA brushes prevents the occurrence of hydrophobic van der Waals interactions and favors association with water molecules. This phenomenon regulates interfacial physicochemical properties that are highly relevant for technological applications and for the design of nanobiointerfaces, including hydration of brushes, adhesion, lubrication, and biopassivity.

More generally, dispersity *within* brushes appears to be a major factor in determining their properties when swollen in aqueous media, in addition to their composition, molar mass, and surface coverage.

This represents a fundamental result especially in the case of brushes comprising a PMA backbone and oligomeric side segments, which are already broadly employed in materials science and nanotechnology and feature a structure reminiscent of a large array of biomacromolecules.

In contrast to their natural “analogues”, which are intrinsically characterized by high purity and structural discreteness, synthetic brushes are often polydisperse, and side-chain dispersity emerges as one of the main tuning parameters for their properties.

These findings open up previously unknown possibilities in the design of polymer adsorbates for the functionalization of biomaterials, whose main-chain and side-chain dispersities can nowadays be precisely tailored by employing controlled-polymerization techniques.

## Methods

### Materials

2-Ethyl-2-oxazoline (EOX, 99%, Sigma-Aldrich) was distilled over KOH to remove traces of water. Methyl *p*-toluenesulfonate (MeOTs, 98%, Sigma-Aldrich) was purified by distillation under reduced pressure over CaH_2_, and methacrylic acid (MA, 99%, Sigma-Aldrich) was purified by filtration on a basic alumina column before use. Triethylamine (TEA, ≥99.5% Sigma-Aldrich) was purified by distillation over KOH. 4-Cyano-4-[(dodecylsulfanylthiocarbonyl)sulfanyl]pentanoic acid (CDPA, 97%, Sigma-Aldrich) was used as received. Azobisisobutyronitrile (AIBN, 98%, Sigma-Aldrich) was recrystallized from methanol. Toluene was refluxed over CaH_2_ for 4 h under nitrogen atmosphere and distilled. Sodium carbonate (NaHCO_3_, ≥99.5%), magnesium sulfate (MgSO_4_, anhydrous, ≥99.5%), potassium hydroxide (KOH, ≥98.5%), calcium hydride (CaH_2_, 95%), acetonitrile (ACN, 99.9+% dry with molecular sieves), methanol (MeOH, >99.9% HPLC grade), and *N*,*N*-dimethylformamide (DMF, >99%) were purchased from Sigma-Aldrich and used as received. (±)-α-Lipoic acid (>98%) was purchased from Acros Organics.

### Synthesis of OEOX_P_MA

To an oven-dried 100 mL Schlenk flask were added dry ACN (20 mL) and EOX (10 g, 101 mmol, 4 equiv) under nitrogen. Then MeOTs (4.7 g, 25 mmol, 1 equiv) was added at 0 °C under nitrogen and stirred for another 15 min before the polymerization mixture was heated to 70 °C and stirred for 15 h under argon. After this time, the polymerization was terminated by adding a solution of MAA (4.3 g, 50 mmol, 2 equiv) and TEA (5.1 g, 50 mmol, 2 equiv) in dry ACN (10 mL) at room temperature and left stirring for another 24 h under argon at 60 °C. MAA was previously filtered through a basic alumina plug before use. The solvent was removed under reduced pressure, and the residue was redissolved in chloroform (250 mL). The organic solution was washed with a saturated solution of NaHCO_3_ (2 × 200 mL) and finally with brine (1 × 200 mL). The organic phase was dried over MgSO_4_, filtered, and the solvent was evaporated under reduced pressure prior to addition of a point of spatula of hydroquinone in order to prevent the possible self-polymerization. The residue was then dissolved in water and freeze-dried in order to obtain a white and very hygroscopic yellowish solid (11.5 g, 92% yield).

The chemical structure and the purity of the synthesized monomer were determined by ^1^H NMR (400 MHz) (Figure S1) by LC–MS and HPLC (Figure 2).

### Purification of OEOX_P_MA into Discrete OEOX_*n*_MA

OEOX_P_MA was dissolved in the minimum amount of DCM. The obtained viscous solution was then purified by flash silica gel column chromatography (proportion between sample and SiO_2_ should lie between 1:70 to 1:100 w/w) using a manually performed gradient of mobile phase from 100% DCM to 90%/10% DCM/MeOH. Single-fraction detection was performed using SiO_2_-coated TLC sheets stained with KMnO_4_ solution. Each fraction was isolated by solvent evaporation under reduced pressure, redissolution in water, freeze-drying, and finally storage below −20 °C prior to use. All fractions were finally characterized by ^1^H NMR (Figures S2–S4) and by LC–MS (Figure 2).

### RAFT Polymerization

POEOX_P_MA and POEOX_*n*_MAs with *n* = 3, 4, and 5 were synthesized by RAFT polymerization with 60:1:0.2 monomer:CDPA:AIBN molar ratio in toluene solution (0.5 M monomer concentration). In a typical procedure, OEOX_3_MA (0.328 g, 0.825 mmol), CDPA (5.55 mg, 0.014 mmol), AIBN (0.45 mg, 0.0027 mmol), and anhydrous toluene (1.65 mL) were loaded in a 10 mL Carius tube, and the mixture was degassed with five freeze–pump–thaw cycles. The polymerization was carried out under vacuum at 70 °C. After 24 h, the reaction was stopped by exposure to air, and the crude product was precipitated into a large excess of *n*-hexane. The final product POEOX_3_MA (conversion 95%) was characterized by ^1^H NMR (^1^H NMR (CDCl_3_) 4.4–3.9 (CH_2_COO), 3.9–3.25 (CH_2_CH_2_N), 3.1–2.9 (CH_3_N), 2.7–2.25 (CH_3_CH_2_CO), 2.20–0.70 (CH_3_CH_2_CO, CH_2_CCH_3_)) and size exclusion chromatography (SEC) (Figure S5).

### Synthesis of *N*-(4-(Aminomethyl)benzyl)-5-(1,2-dithiolan-3-yl)pentanamide (ANPIS)

(±)-α-Lipoic acid (2.00 g, 9.70 mmol, 1 equiv) was dissolved in 12 mL of anhydrous chloroform. 1,1-Carbonyldiimidazole (2.00 g, 12.3 mmol, 1.3 equiv) was added to the lipoic acid solution and stirred for 5 min at room temperature. The resultant solution was added dropwise into a 1,4-benzenedimethanamine suspension (6.6 g, 48.5 mmol, 5 equiv) in dry chloroform (8 mL) and stirred for 40 min in an ice bath and for another 3 h at room temperature. The crude product was washed three times with 20 mL of 10% NaCl aqueous solution and once with 20 mL of water. It was dried with sodium sulfate and the solvent removed using a rotary evaporator. The residue was purified by flash silica gel chromatography using 80/20 CHCl_3_/MeOH + 1% TEA as mobile phase. The product (ANPIS) was obtained as a beige solid (2.3 g, 7.3 mmol, 75% yield). ^1^H NMR (Figure S6, 400 MHz, DMSO-*d*_6_) δ = 8.34–8.24 (m, 1H), 7.34–7.10 (m, 4H), 4.22 (d, *J* = 5.9 Hz, 2H), 3.73 (s, 2H), 3.59 (dq, *J* = 8.6, 6.2 Hz, 1H), 3.25–3.04 (m, 2H), 2.40 (dq, *J* = 12.4, 6.1, 5.6 Hz, 1H), 2.13 (t, *J* = 7.3 Hz, 2H), 1.85 (dq, *J* = 13.4, 6.8 Hz, 1H), 1.76–1.58 (m, 1H), 1.54 (qd, *J* = 9.5, 8.0, 3.1 Hz, 3H), 1.45–1.21 (m, 2H) ppm. ^13^C NMR (100 MHz, DMSO-*d*_6_) δ = 171.85, 140.62, 138.03, 127.26, 127.03, 56.10, 44.70, 41.74, 38.08, 35.13, 34.09, 28.29, 25.04 ppm.

### Synthesis of POEOXMA-ANPIS Conjugates

The synthesis of POEOX_3_MA-ANPIS conjugate was exemplarily reported. POEOX_3_MA (227 mg, 0.02 mmol of free acid group), ANPIS (65 mg, 0.2 mmol, 10 equiv), and 1-[(1-(cyano-2-ethoxy-2-oxoethylideneaminooxy)dimethylaminomorpholino)]uronium hexafluorophosphate (COMU) (85 mg, 0.2 mmol, 10 equiv) were dissolved in 4 mL of DCM in a 10 mL Schlenk flask. The flask was purged with a stream of nitrogen, and DIPEA (100 mL, 0.3 mmol, 30 equiv) was finally added and the reaction mixture stirred at RT for 48 h under argon. Then 7 mL of MeOH was added to the reaction mixture, and the flask was washed with a further 8 mL of MeOH and finally purified by dialysis (1 kDa MWCO) in MeOH for 48 h. The solvent was removed to yield POEOX_3_MA-ANPIS as a yellow, viscous polymer. The chemical structure and the purity of POEOX_P_MA-, POEOX_3_MA-, POEOX_4_MA-, and POEOX_5_MA-ANPIS were determined by ^1^H NMR (400 MHz) (Figures S7–S10).

### Surface Assembly of POEOXMA-ANPIS Adsorbates on Au

Silicon wafers coated with a 100 nm thick Au layer were prepared by reactive magnetron sputtering (Paul Scherrer Institute, Villigen, Switzerland). The substrates (10 × 20 mm^2^) were cleaned for 1 min in piranha solution (3:1 mixture of concentrated H_2_SO_4_ and H_2_O_2_) and later were extensively washed with ultrapure water and absolute ethanol. Surface assembly of POEOXMA-ANPIS adsorbates was performed by immersing the Au-coated substrates for 2 h in 1 mg mL^–1^ methanolic solutions at room temperature (RT). The functionalized samples were subsequently rinsed with ultrapure water and absolute ethanol to remove physisorbed species and finally dried under a stream of N_2_.

### NMR Spectroscopy

^1^H and ^13^C NMR spectra were recorded on a Bruker Avance III HD 400 MHz spectrometer at room temperature using D_2_O, CDCl_3_, or DMSO-*d*_6_ as solvents.

### Size Exclusion Chromatography (SEC)

SEC was performed by using a Viscotek gel permeation chromatography (GPC) system (Malvern, Worcs, U.K.) equipped with a pump and degasser (GPCmax VE2001, 1.0 mL min^–1^ flow rate), a detector module (Viscotek 302 TDA), and three columns (2× PLGel Mix-C and 1× ViscoGEL GMHHRN 18055, dimensions 7.5 mm × 300 mm for each column) using THF as eluent. Each sample was prepared dissolving the polymer at a defined concentration of 1 mg mL^–1^ in THF containing 0.3% of toluene.

### Contact Angle (CA) Measurements

Surface wettability was determined by static and dynamic CA (DSA 100, Krüss, Hamburg, Germany) in an automated procedure.

Static CA measurements were performed using the sessile drop technique. A sessile drop was deposited onto the brush-functionalized Au surfaces with the aid of an automated syringe, and the drop contour was fit by the Young–Laplace method. Twenty measurements were recorded for each type of sample.

In order to record the values of θ_A_ and θ_R_, the volume of ultrapure water drops dispensed on the brush-functionalized substrates was progressively increased and decreased from 4 to 10 μL at a speed of 15 μL min^–1^. Three different locations were measured on each sample.

### Variable-Angle Spectroscopic Ellipsometry (VASE)

Ex situ VASE measurements were carried by using a variable-angle spectroscopic ellipsometer (M-2000F, Woollam Co., Inc., Lincoln, NE, USA) to determine the values of *T*_dry_ of the polymer brushes assembled on Au. The measurements were performed in the spectral range of 290–900 nm using focusing lenses at three different angles of incidence (60°, 65°, and 70°) from the surface normal. Each data point resulted from the average of 20 measurements, and the obtained raw ellipsometric data were fitted with a bilayer model (Au and organic adlayer) using the analysis software WVASE32. The thickness of Au layer (100 nm) was assumed to be constant. The *n* and *k* values for Au were fitted by measuring a freshly cleaned Au substrate (without brushes), and the organic adlayer (polymer brush) was fitted using the Cauchy model (*A_n_* = 1.45 and *B_n_* = 0.01, *C_n_* = 0). A homogeneous mass distribution of the organic adlayer perpendicular to the Au surface was assumed with a density of 1.14 g cm^–3^ for the polymer brush.^64^

### Quartz Crystal Microbalance with Dissipation (QCMD)

QCMD experiments were performed at ambient temperature, using a Q-Sense E4 (Q-Sense AB, Göteborg, Sweden) equipped with dedicated Q-Sense AB software. Au-coated crystals (LOT-Oriel AG) with a fundamental resonance frequency of 5 MHz were used as substrates. Before the experiment, the substrates were sonicated twice in toluene and twice in isopropanol and finally subjected to UV–ozone (UV Clean model 135500 from Boekel Industries, Inc.) for 30 min. Au-coated crystals were first exposed to ultrapure water until a stable baseline was recorded (Figure S11). Water was subsequently replaced with methanol until a new baseline was reached. A 1 mg mL^–1^ ethanolic solution of POEOXMA-ANPIS was then injected until complete formation of POEOXMA brushes was accomplished. Removal of physisorbed polymer adsorbates was performed by rinsing with ethanol and finally with ultrapure water. The values of *T*_wet_ of POEOXMA brushes were obtained by applying an extended viscoelastic model (Qtools 3 software), fitting the frequency (Δ*F*) and dissipation shifts (Δ*D*) recorded after the formation of POEOXMA brush layers and their swelling in ultrapure water, using four different overtones (3^rd^, 5^th^, 7^th^, and 9^th^) for each sample (see Supporting Information). Two crystals for each film were used to calculate the mean values of *T*_wet_ and standard deviations. Fixed parameters for the fitting were fluid density (997 kg m^–3^), layer density (1100 kg m^–3^), and fluid viscosity (0.009 kg m^–1^ s^–1^). The parameters that were fitted (while being constrained to physically meaningful boundaries) were the layer’s viscosity (0.009–0.05 kg m^–1^ s^–1^), shear modulus (10^4^–10^8^ Pa), *T*_wet_ (10^–9^–10^–8^ m), and hydrated mass.

All QCMD experiments were conducted at ambient temperature, which oscillated between 20 and 22 °C.

### UV/Visible Spectroscopy

Thermoresponsive behavior of POEOXMAs in solution was analyzed by performing turbidity measurements using a Shimadzu (Kyoto, Japan) 2450 UV/vis spectrometer equipped with a S-1700 thermoelectric single cell holder. 5 mg mL^–1^ aqueous solution of the POEOXMAs was analyzed in quartz cuvettes with a 10 mm optical path. Transmittance was measured at a fixed wavelength of 700 nm. Turbidity measurement were carried out in the temperature range 25–95 °C. Cloud point temperatures (*T*_cp_) were assigned at temperatures that showed a 50% decrease in transmittance with respect to the value recorded at 25 °C.

### Atomic Force Microscopy (AFM)

Lateral and normal force measurements were performed by using MFP3D AFM (Asylum Research, Oxford Instruments, Santa Barbara, USA) under 1 mM (4-(2-hydroxyethyl)-1-piperazineethanesulfonic acid) (HEPES) buffer solution at pH = 7.4. Colloidal AFM probes were prepared by attaching a ∼20 μm diameter silica bead (EKA Chemicals AB, Kromasil R, Sweden) onto tipless cantilevers (CSC38-C/tipless/Cr-Au, Mikromasch, Bulgaria). Colloidal AFM probes were subsequently covered by a 3 nm thick W layer, followed by a 20 nm thick layer of Au by a metal evaporator (MED020 coating system, BAL-TEC, Balzers, Lichtenstein). POEOXMA brushes with different side-chain dispersity and length were later grafted to the colloidal probes following the same procedures applied for the flat Au-coated substrates. Four different cantilevers having nearly identical normal and lateral spring constant values were selected for preparing colloidal probes with four different side-chain dispersity.

The normal (*K*_N_) and torsional (*K*_T_) spring constant values for all the cantilevers were obtained by thermal noise^65^ and Sader’s method^66^ prior to the attachment of the colloids. The obtained *K*_N_ and *K*_T_ values for four different cantilevers were reported in Table S3.

In all the AFM measurements, the diameter of the Au-coated, silica colloidal probe was ∼20 μm. Friction measurements were performed by obtaining 5–6 “friction loops” along the same line for each applied load over 3 different positions on each sample (scan rate of 0.5 Hz, sliding distance of 5 μm), from which the average friction forces and the standard deviations were calculated. The friction force calibration was carried out by using the method following Cannara et al.^67^ Each set of FS curves was obtained over 3 different positions for each sample. A scanning distance of 1 μm and a scanning rate of 0.5 Hz were used.

All AFM experiments were conducted at ambient temperature, which oscillated between 20 and 22 °C.

## Supporting Information Available

SEC elugrams and ^1^H NMR spectra for all compounds, data on yield for purified OEOXMA fractions, and additional data on characterization of POEOXMAs (PDF)
