# Supplementary material for: Dispersity within Brushes Plays a Major Role in Determining Their Interfacial Properties: The Case of Oligoxazoline-Based Graft Polymers
Source: J Am Chem Soc. 2021 Nov 5;143(45):19067–77. doi: 10.1021/jacs.1c08383 (PMC8769490; doi:10.1021/jacs.1c08383)
Supplement: Supplementary file 1 — ja1c08383_si_001.pdf [file ja1c08383_si_001.pdf]

SUPPORTING INFORMATION

Dispersity *Within* Brushes Plays a Major Role in Determining Their Interfacial Properties: The Case of Oligoxazoline-Based Graft Polymers

Matteo Romio,[1,2] *Benjamin Grob,*[2] *Lucca Trachsel,*[3] *Andrea Mattarei,*[4] *Giulia Morgese,*[5] *Shivaprakash N. Ramakrishna,*[6] *Francesca Niccolai,*[7] *Elisa Guazzelli,*[7] *Cristina Paradisi,*[8] *Elisa Martinelli,*[7] *Nicholas D. Spencer,*[2] *Edmondo M. Benetti*[1,2,8]*

1) Biointerfaces Lab, Swiss Federal Laboratories for Materials Science and Technology (Empa), Lerchenfeldstrasse 5, St. Gallen, Switzerland.

2) Laboratory for Surface Science and Technology, Department of Materials, ETH Zürich, Vladimir-Prelog-Weg 5, 8093 Zürich, Switzerland.

3) George & Josephine Butler Polymer Research Laboratory, Department of Chemistry, University of Florida, PO Box 117200, Gainesville, Florida 32611-7200, United States.

4) Department of Pharmaceutical and Pharmacological Sciences, University of Padova Via Marzolo 5, 35131 Padova, Italy.

5) Institute of Materials and Process Engineering (IMPE), School of Engineering (SoE), Zürich University of Applied Sciences (ZHAW), Technikumstrasse 9, 8401 Winterthur, Switzerland.

6) Soft Materials and Interfaces, Department of Materials, ETH Zürich, Vladimir-Prelog-Weg 5, 8093 Zürich, Switzerland.

7) Department of Chemistry and Industrial Chemistry, University of Pisa, Via Moruzzi 13, 56124 Pisa, Italy.

8) Department of Chemical Sciences, University of Padova, Via Marzolo 1, 35131 Padova, Italy.

Email: edmondo.benetti@unipd.it

Fraction	DP (^{1}H-NMR)	Isolated weight (g)	Recovered amount (w%)	Recovered amount (mol%)
**OEOX$_2$MA**	2	0,19	3	4
**OEOX$_3$MA**	3	1,09	12	18
**OEOX$_4$MA**	4	1,48	16	20
**OEOX$_5$MA**	5	2,16	24	23
**OEOX$_6$MA**	6	1,99	22	18
**OEOX$_7$MA**	7	1,16	13	10
**OEOX$_8$MA**	8	0,94	10	7

Table S1. Fractions of OEOX$_n$MA macromonomers isolated following flash column chromatography performed from 10 g of polydisperse OEOX$_P$MA. Around 1 g of polydisperse macromonomer could not be recovered presumably due to strong interactions with the silica column.

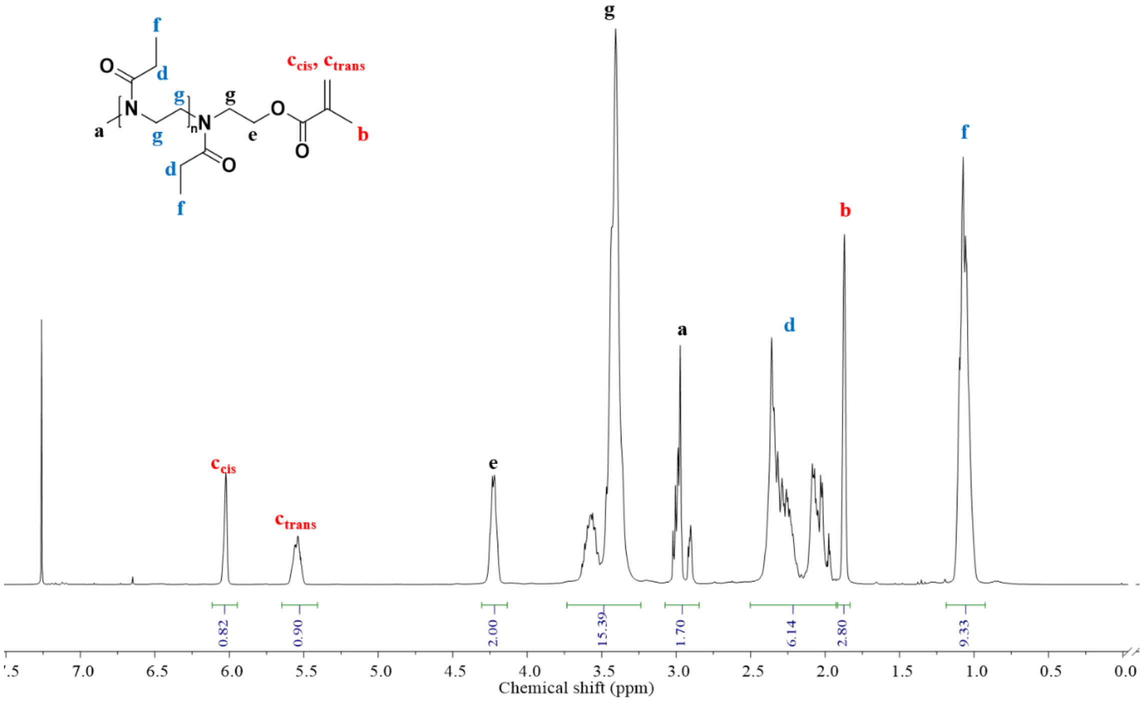

Figure S1. ^{1}H-NMR (400 MHz) spectrum of OEOX$_P$MA recorded in $CDCl_3$.

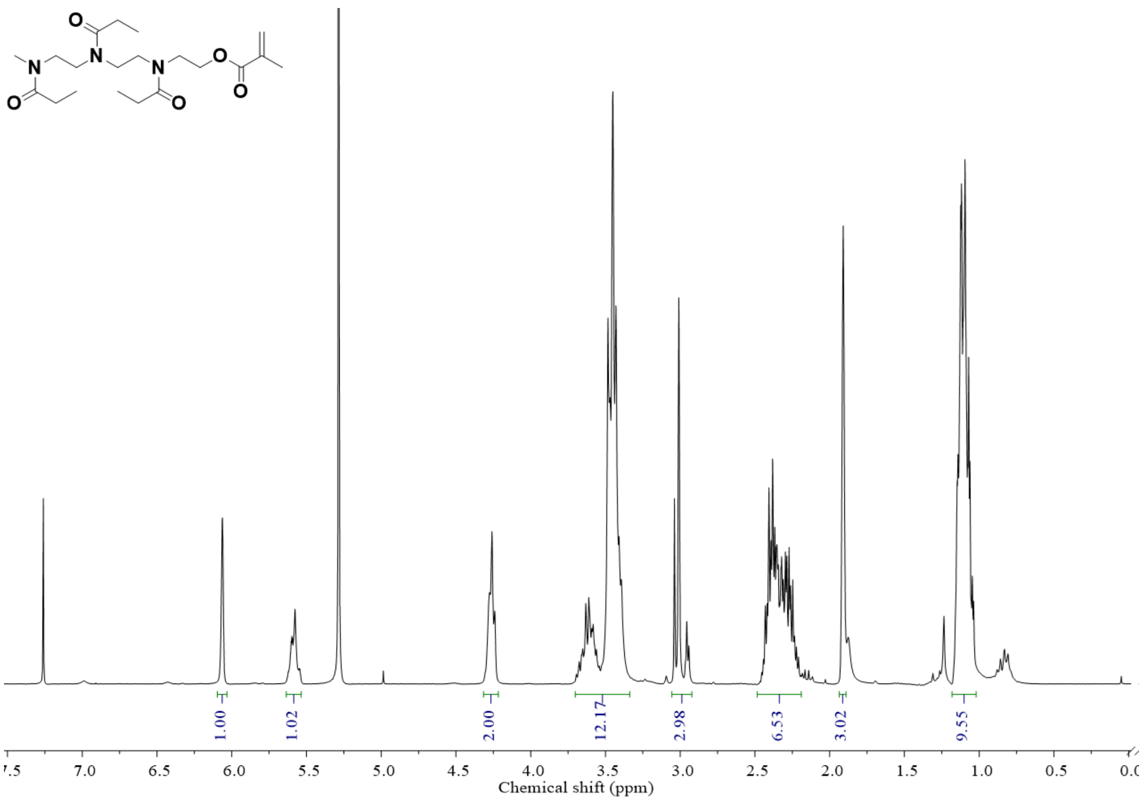

Figure S2. ^{1}H-NMR (400 MHz) spectrum of $OEOX_3MA$ recorded in $CDCl_3$.

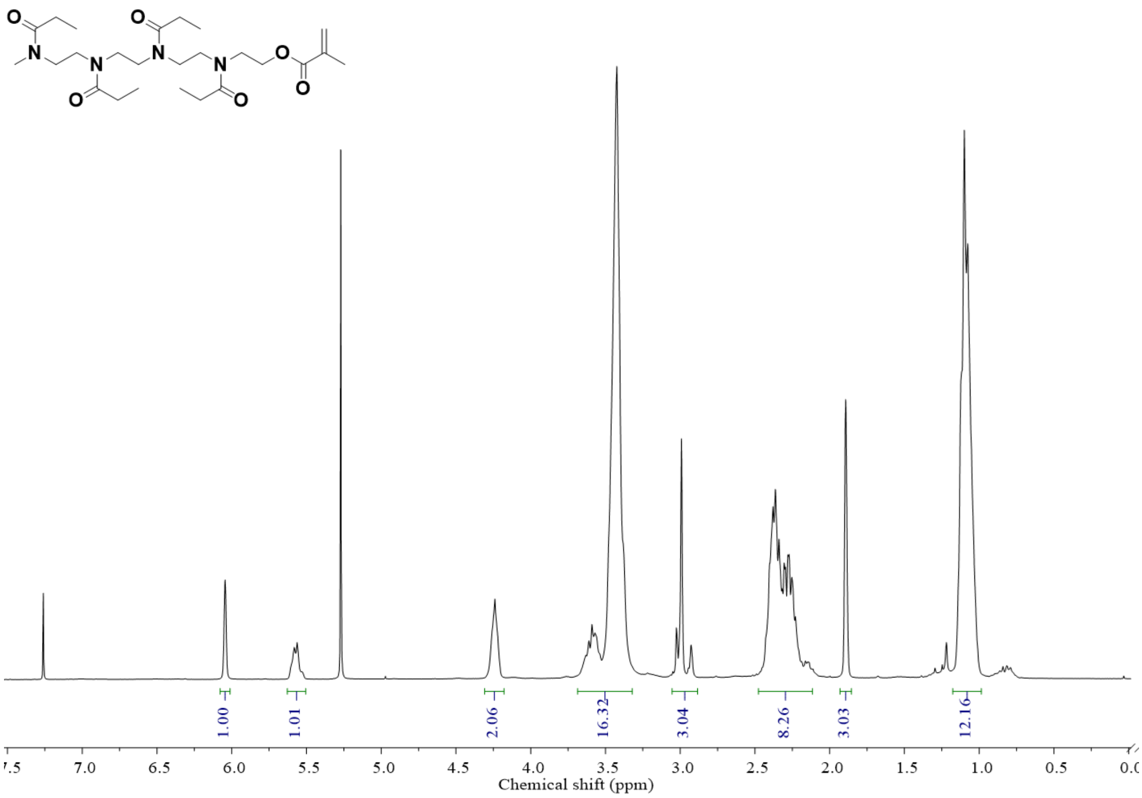

Figure S3. ^{1}H-NMR (400 MHz) spectrum of $OEOX_4MA$ recorded in $CDCl_3$.

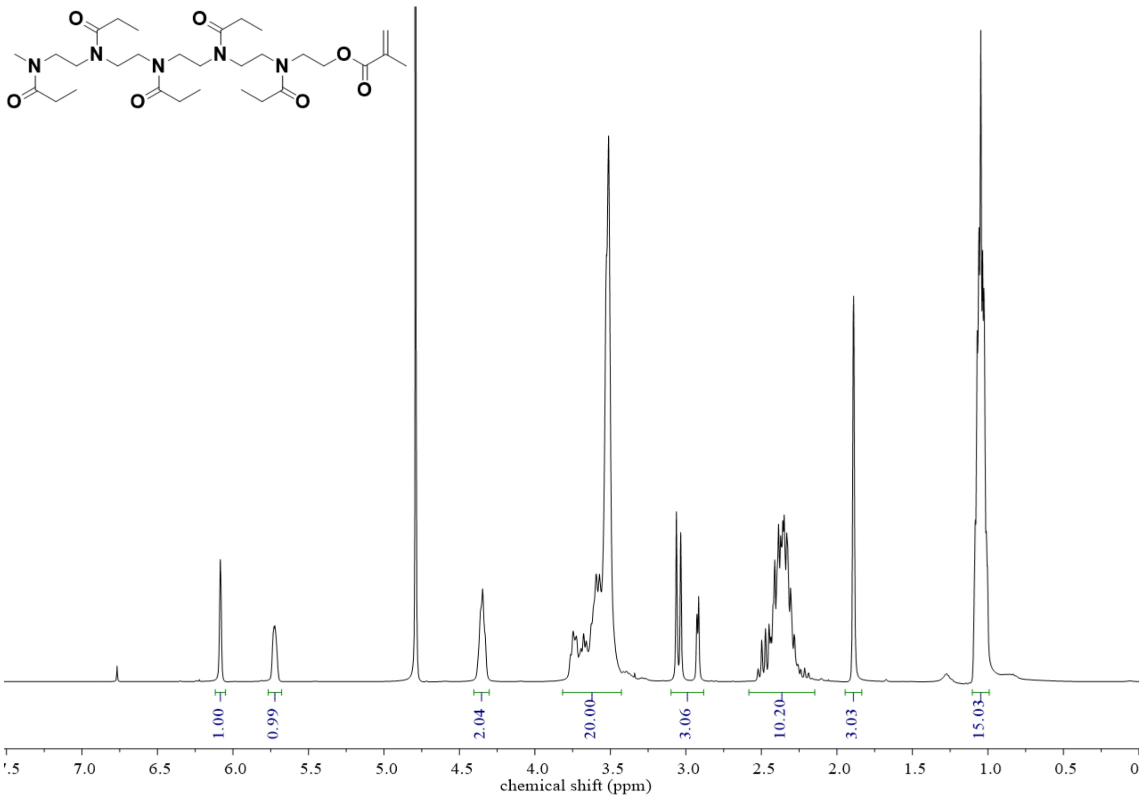

Figure S4. ^{1}H-NMR (400 MHz) spectrum of OEOX$_5$MA recorded in D_2O.

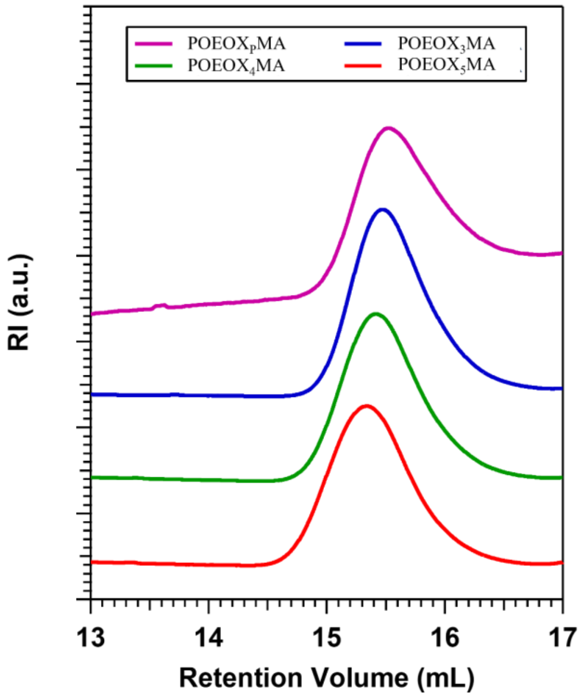

Figure S5. SEC elugrams of POEOX$_P$MA, POEOX$_3$MA, POEOX$_4$MA and POEOX$_5$MA recorded using THF as eluent.

polymer	*Conv* %	M_n (theor.) [kDa]	DP$_n$ (theor.)	M_n (SEC) [kDa]	DP$_n$ (SEC)	M_n (NMR) [kDa]	DP$_n$ (NMR)
**POEOX$_P$MA**	98	29.2	58.8	10.8	24.0	22.8	50.7
**POEOX$_3$MA**	95	22.7	57.0	11.5	28.9	25.1	63.1
**POEOX$_4$MA**	98	29.2	58.8	15.6	31.4	24.9	50.1
**POEOX$_5$MA**	96	34.3	57.6	20.9	35.1	16.1	27.0

Table S2. Values of M_n and DP for the different POEOXMA samples estimated by measuring conversion, SEC and NMR.

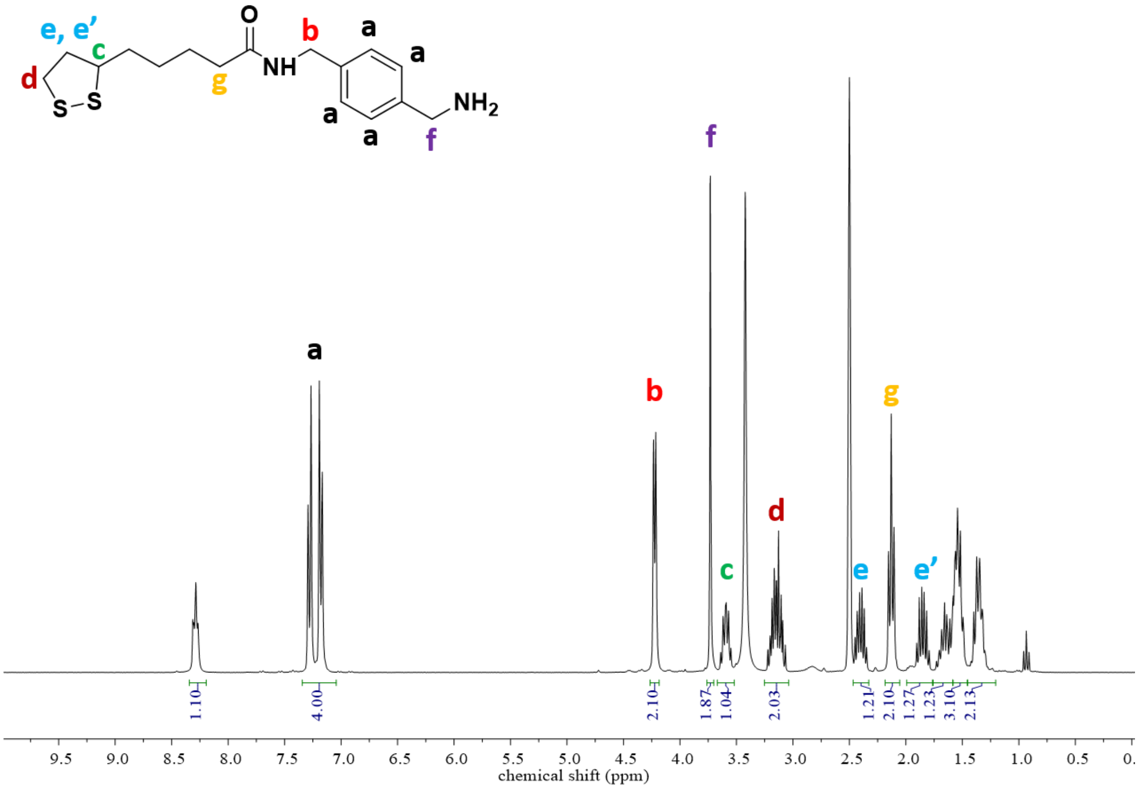

Figure S6. ^{1}H-NMR (400 MHz) spectrum of N-(4-(aminomethyl)benzyl)-5-(1,2-dithiolan-3-yl)pentanamide (ANPIS) recorded in DMSO-d$_6$.

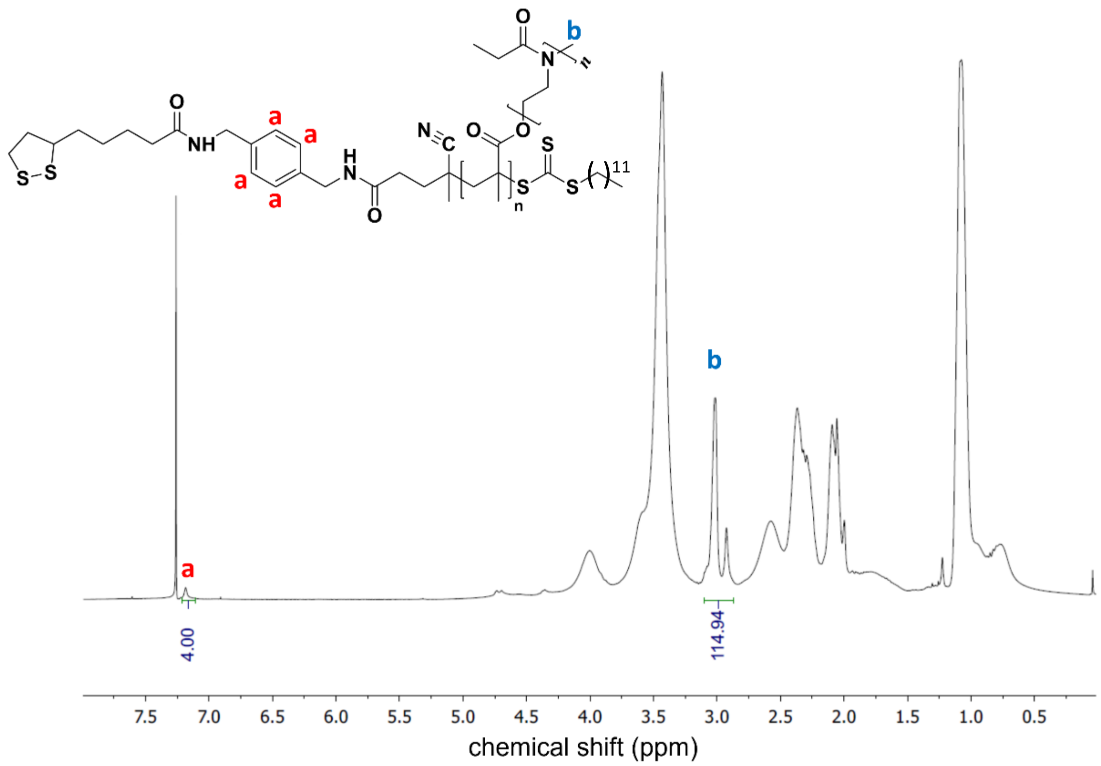

Figure S7. ^{1}H-NMR (400 MHz) spectrum of POEOX$_P$MA-ANPIS conjugate recorded in $CDCl_3$.

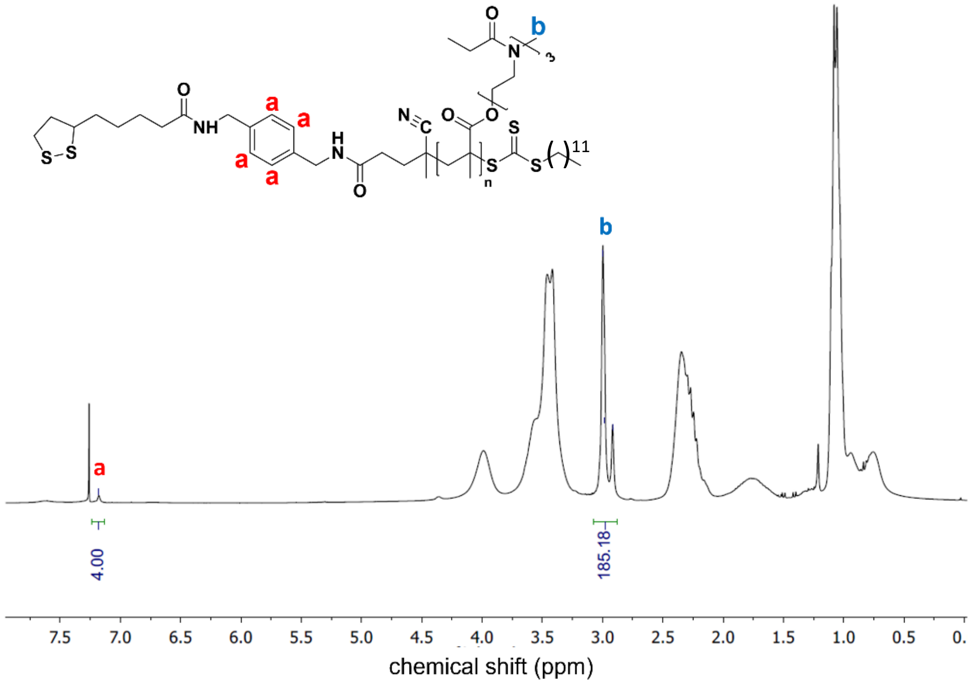

Figure S8. ^{1}H-NMR (400 MHz) spectrum of POEOX$_3$MA -ANPIS conjugate recorded in $CDCl_3$.

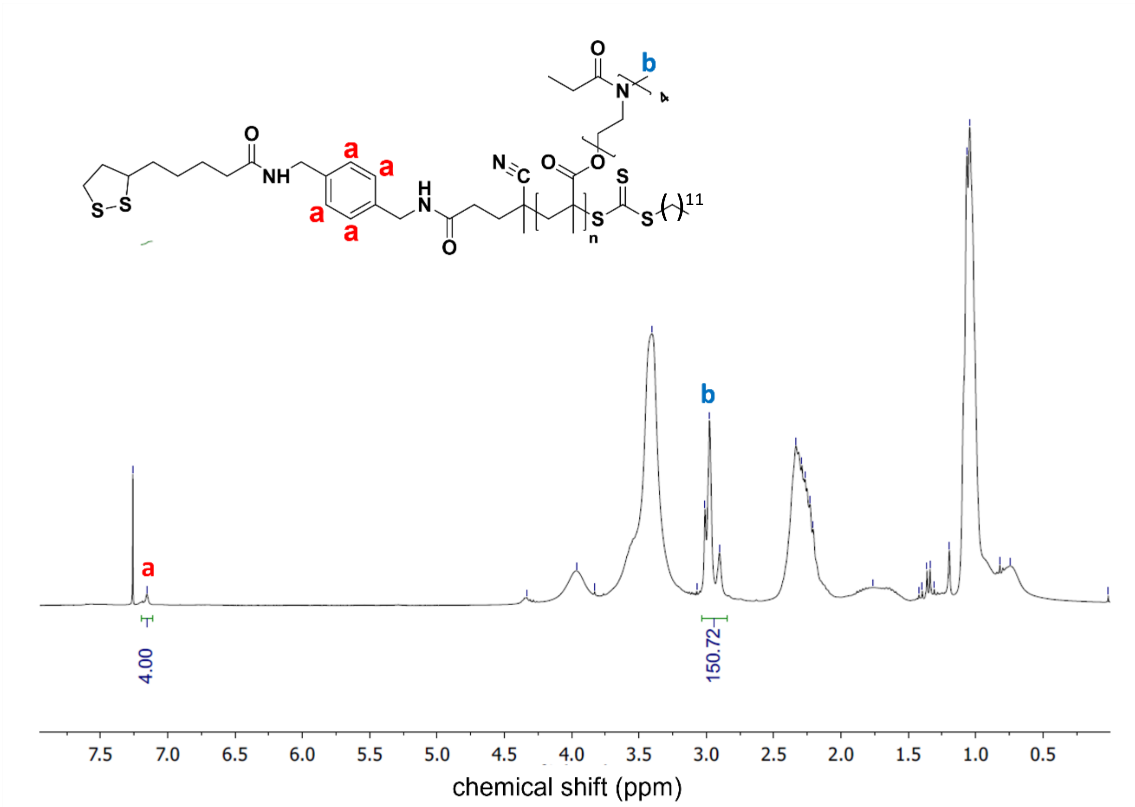

Figure S9. ^{1}H-NMR (400 MHz) spectrum of POEOX$_4$MA-ANPIS conjugate recorded in $CDCl_3$.

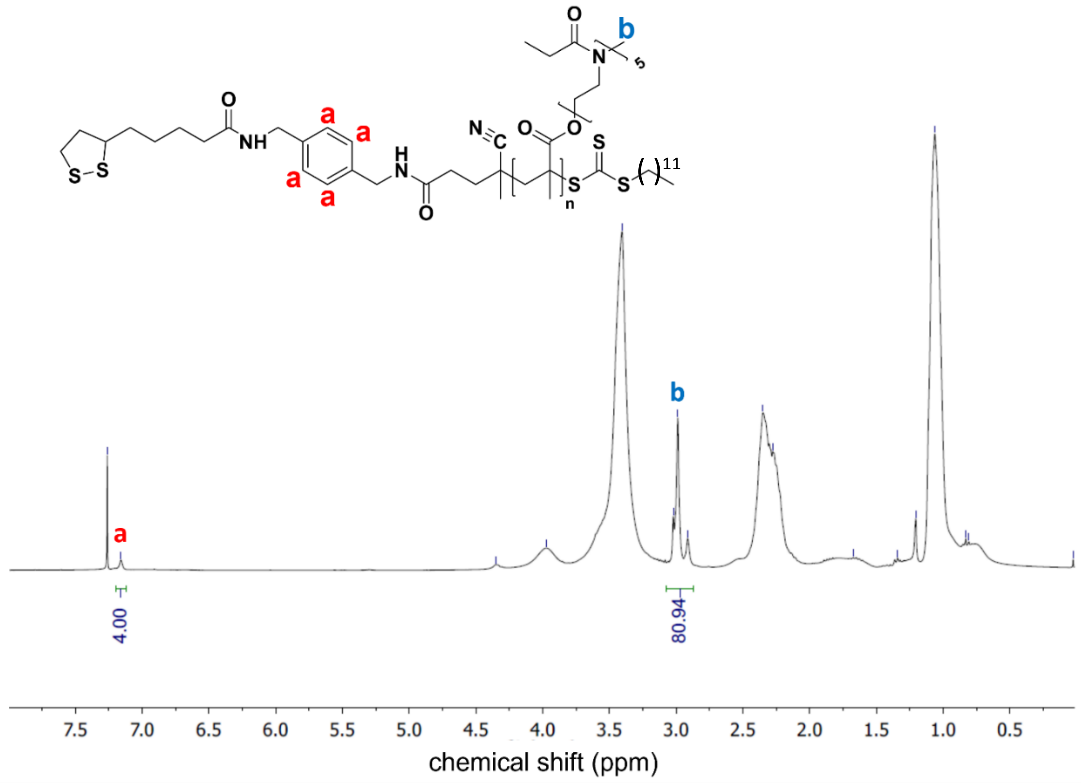

Figure S10. ^{1}H-NMR (400 MHz) spectrum of POEOX$_5$MA-ANPIS conjugate recorded in $CDCl_3$.

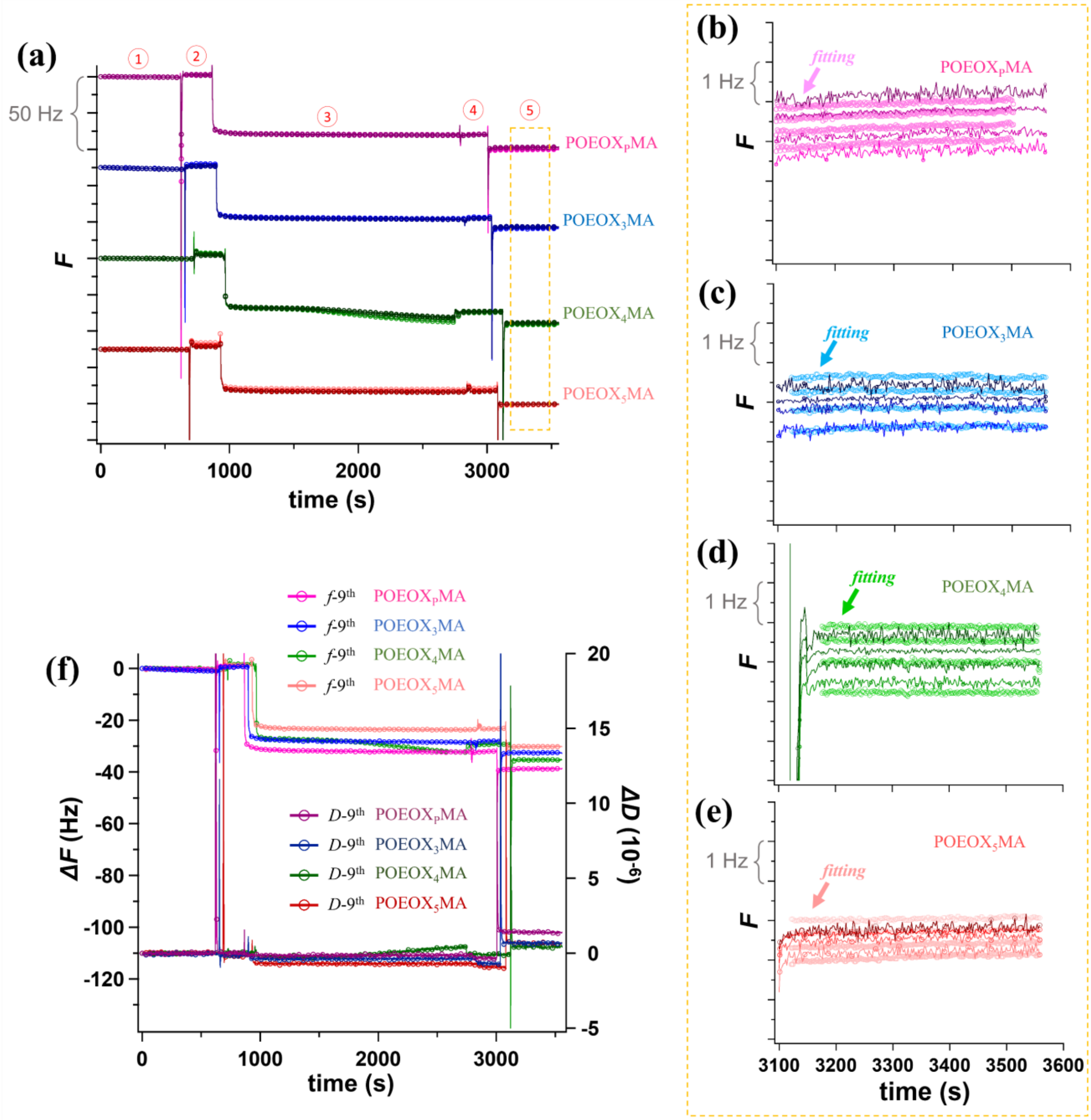

Figure S11. (a) QCMD sensograms displaying frequency shifts (3rd, 5th, 7th and 9th overtones) for each POEOXMA brush. Au-coated QCMD sensors were first incubated in ultrapure water (1) until a stable baseline as recorded. Ethanol was subsequently injected (2) followed by 1 mg mL^{-1} solution of POEOXMA (3). After polymer adsorption, the formed brush layers were rinsed with ethanol (4), and finally equilibrated in ultrapure water (5). ΔF and ΔD shifts recorded when POEOXMA brushes were swollen in ultrapure water (regions within the dashed area) were fitted with a Voigt extended viscoelastic model in order to obtain the values of T_{wet}. (b-e) Representative fittings for each POEOXMA brush. (f) ΔF and ΔD shifts (9th overtone) obtained during the formation of POEOXMA brushes.

Polymers coated cantilevers	K_N ($N\ m^{-1}$)	K_T ($N\ m$)
POEOX$_p$MA	0.095	2.45 E-9
POEOX$_3$MA	0.107	2.84 E-9
POEOX$_4$MA	0.106	2.90 E-9
POEOX$_5$MA	0.105	3.01 E-9

Table S3. Normal (K_N) and torsional (K_T) spring constant values of the cantilevers used for AFM experiments.

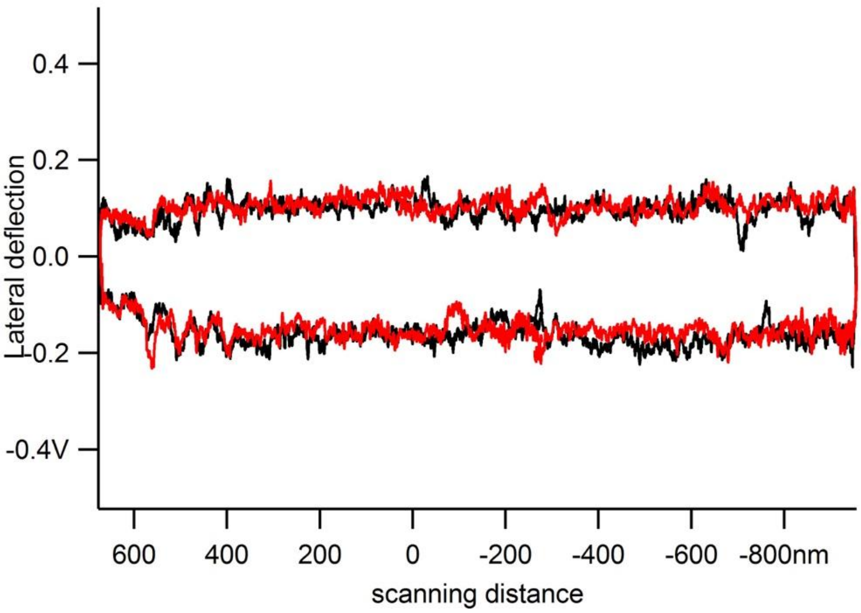

Figure S12. Friction "loop" obtained by laterally scanning POEOX$_3$MA brushes while applying 4 nN of normal load. The red curve corresponds to the 1st scan, the black curve corresponds to the 6th scan.
